# Beyond rejection: engineering immune responses to redefine skin graft survival

**DOI:** 10.3389/fimmu.2026.1780278

**Published:** 2026-03-10

**Authors:** Daniela Grinis, Jacob Bouzaglou, Anish R. Maskey

**Affiliations:** 1 Department of Basic Science, Touro College of Osteopathic Medicine, Great Falls, MT, United States; 2 Department of Basic Science, Touro College of Osteopathic Medicine, Harlem, NY, United States; 3 Department of Pathology, Microbiology & Immunology, New York Medical College, Valhalla, NY, United States

**Keywords:** biomaterials, immunomodulation, inflammation, regenerative medicine, skin graft rejection, tissue engineering

## Abstract

Skin grafting remains a fundamental reconstructive technique in reconstructive and dermatologic surgery for restoring skin integrity following burns, trauma, chronic ulcers, and excision of malignancies. However, a graft survival rate is determined by multiple factors and by a delicate balance between inflammatory and reparative immune system processes. Controlled inflammation promotes wound healing while excessive or prolonged immune activation has the potential to result in cytotoxicity and complement activation which will eventually lead to graft rejection. Early rejection is driven by antibody-meditated complement cascade and innate effector cells like macrophages and natural killer (NK) cells while long-term graft rejection often results from T cell-mediated cytotoxicity and alloantibody production. Molecular mediators such as reacting oxygen species, pro-inflammatory cytokines, and matrix metalloproteinases (MMP) further amplify tissue injury and prevent healing. Current immunosuppressive therapies like calcineurin inhibitors and different corticosteroids reduce rejection to a certain level and are limited by systemic toxicity and impaired wound healing. Emerging strategies including bioengineered scaffolds, localized cytokine inhibitors, and stem cell-derived factors offer targeted immunomodulation to enhance graft tolerance and long-term survival. Despite promising preclinical data, translation into humans and application of the new emerging therapies remain constrained by species differences, ethical considerations, and variability in patient immune profiles. Advancing humanized models, biomaterial-based therapies, and precision immunotherapy will be pivotal in optimizing graft outcomes. This review aims to highlight how strategic modulation of inflammatory pathways can predict better graft outcomes and pave the way for next-generation regenerative therapies.

## 1 Introduction

Skin grafting is a foundational technique in both plastic and reconstructive surgery as well as dermatology, frequently used to restore skin integrity following burns, trauma, chronic ulcers, and excision of cutaneous malignancies (1). Worldwide, deep dermal and full-thickness thermal burns represent the most common reason for skin grafting (2). In the United States, burn injuries place a significant economic and clinical burden on both the patients and the healthcare system. Analysis of commercial insurance claims to identify 63,540 burn patients, of whom 4.2% required inpatient admission within 30 days, representing the population most likely to undergo operative wound closure such as split-thickness skin grafting. Even in cases directed exclusively in the outpatient setting, burn case generated substantial cumulative costs, with mean expenditures of $2,896 at 30 days, and continued financial impact extending for up to three years following the initial injury (3). These findings highlight that burns requiring inpatient care- particularly those requiring grafting - represent a disproportionately resource intensive subset of patients, emphasizing the importance of optimizing graft success and minimizing complications. According an observational study done at the Al-Gumhouri Hospital Burn and Plastic Surgery Center, 198 patients undergoing split-thickness skin grafts (STSGs), burns accounted for 65.2% of skin defects requiring grafting, followed by traumatic wounds (21.7%), postsurgical excisions (7.1%), necrotizing infection (3%), and tumor resection (3%) (4). According to the World Health Organization (WHO), 180,000 deaths each year are attributed to burns, while non-fatal burn injuries, approximately 11 million annually, are the leading cause of prolonged hospitalization, disability, and long-term psychological affect (5).

Preclinical models have played a critical role in uncovering the immunologic mechanisms underlying skin graft rejection. *In vitro* studies utilizing co-cultures of donor antigen-presenting cells and recipient T lymphocytes has provided detailed insights into the pathways of alloantigen recognition, T cell activation, and cytokine signaling (6). Meanwhile, murine models of skin transplantation have enabled precise investigation of both innate and adaptive immune responses, including the kinetics of complement cascade activation, cytotoxic T lymphocyte infiltration and the tolerogenic effects of regulatory T cells (7). Such models allow for genetic manipulation (e.g., knockout mice) to dissect the role of individual molecules like interleukin-2 (IL-2) or major histocompatibility complex (MHC) class I/II complexes in elucidating rejection mechanisms. Nevertheless, the application to human grafting is still restricted by significant disparities in immunogenetic diversity, skin structure, and immune cell diversity (8). Murine skin differs from human skin in thickness, hair density, and resident immune cell distribution, which can change both antigen presentation and inflammatory profiles. Additionally, human patients often present with comorbidities such as diabetes or immunosuppression, which are difficult to replicate in animal models. While *in-vivo* murine and *in vitro* models remain essential for mechanistic insights, careful interpretation is required when applying these findings to clinical practice.

The success of the graft being “taken” depends on the interplay between many factors such as inflammation, neovascularization, and immune response. While controlled inflammation is essential for wound healing, excessive or prolonged immune response can result in graft rejection and failure (1). The “take” rate of STSGs is approximately 70-90%, although it depends on the center the graft harvest is being done at (67% success rate is more common to resource limited centers). Post-operative infection, hematoma formation, poor perfusion of the wound bed, as well as patients factors (diabetes, malnutrition, smoking) all remain key predictors to a successful graft procedure (2). Across medical specialties, patients frequently present with underlying inflammatory skin conditions, systemic immunosuppression, or extensive tissue injury- all of which can complicate wound healing and negatively impact graft outcomes (9). A thorough understanding of these immunologic processes is critical for optimizing graft survival and improving patient outcomes. The outcome of a skin graft depends on a delicate balance between pro-inflammatory and reparative immune processes. Controlled inflammation is vital for angiogenesis and tissue repair, but when excessive, the same pathways can trigger cytotoxicity, complement activation, and graft rejection. This dual role of inflammation remains a key challenge in optimizing graft survival. Therefore, this review elucidates why optimizing key inflammatory and immunoregulatory pathways is essential- not only to prevent rejection, but also to enhance vascularization, restore normal tissue function and improve overall functional outcomes.

## 2 Immunologic mechanisms underlying graft acceptance and rejection

The early immune response plays a critical role in the initial stages of skin graft rejection. Skin graft outcomes are governed by coordinated interactions between innate and adaptive immune pathways that determine whether a graft is accepted or rejected (2). Early inflammatory responses initiated by innate immune cells are necessary for angiogenesis and tissue repair; however, excessive or prolonged immune activation can shift the wound environment toward cytotoxicity and graft failure (10, 11). In addition to immune signaling, intrinsic properties of the donor tissue- such as antigen composition, cellular content, and graft thickness- further influence graft immunogenicity and survival (2). Full-thickness grafts, which retain dermal antigen-presenting cells such as Langerhans cells, are therefore more immunogenic than split-thickness grafts that contain fewer donor immune cells (12). Optimization of inflammatory and immunoregulatory pathways is thus essential not only to prevent rejection but also to promote vascularization, restore tissue function, and improve clinical outcomes. Upon initial grafting, the innate immune mechanisms are activated usually within minutes to hours, specifically in hyperacute rejection scenarios. This form of graft rejection usually occurs due to preformed recipient antibodies directed against donor human leukocyte antigen (HLA) molecules or ABO blood group antigens (10). The antibodies bind to the graft endothelium surface, activating the classical complement pathway and initiating a cascade of inflammatory reaction, resulting in rapid thrombosis and graft ischemia. This type of reaction is mediated by a type II hypersensitivity response where the humoral mechanism triggers small vessel thrombosis and necrosis. Additionally, innate immune cells such as macrophages and NK cells may be rapidly recruited through cytokine release promoting inflammation and contributing to graft injury and necrosis (10, 11). Therefore, the innate immune system serves as a critical initiator of the immunologic cascade that can progress to acute and chronic rejection if not properly controlled. The adaptive immune system plays a pivotal role in skin graft rejection through T cell-mediated recognition of donor antigens. The response begins with alloantigen recognition *via* two pathways: direct recognition, where recipient CD4^+^ T cells bind to donor MHC class II molecules on donor antigen-presenting cells (APCs) (13), and indirect recognition, where host APCs present processed donor peptides and elicit a CD4^+^ T cell response (14). The direct recognition pathway activates Th1 differentiation and interferon-gamma (IFN-γ) cytokine production, which activates macrophages as well as recruitment of other proinflammatory molecules (6). CD8^+^ T cells are activated by donor MHC class I which contribute to direct cytotoxicity against grafted tissues (7). These T cell-mediated responses are central to acute cellular rejection, which also is an example of a type IV hypersensitivity reaction. Additionally, indirect antigen recognition promotes B cell activation and alloantibody production, contributing to acute humoral rejection. In the indirect response, host APCs process the donor MHC molecule, presenting the internalized peptides on self MHC class II to recipient CD4^+^ T cells. The activated CD4^+^ T cells provide the necessary costimulatory signal to B cells that have internalized the same alloantigen, leading to their differentiation into plasma cells capable of producing donor-specific antibodies (DSAs). These drives complement activation, endothelial injury, as well as vascular inflammation which are all characteristics of acute antibody-mediated rejection (ABMR). This mechanism bridges the cellular and humoral immune responses, providing the indirect pathway as the major drive of chronic graft injury (15). Full-thickness skin grafts (FTSGs) contains complete dermis and epidermis, including resident immune cells such as Langerhans cells, making the grafts more immunogenic (2). However, split-thickness skin grafts (STSGs) include only the epidermis and a partial dermis, reducing donor antigen presentation and therefore leading to lower antigenic stimulation (16). These variations in immune recognition affect the likelihood of rejection and the choice of grafting strategy, especially in patients with overactive immune systems.

**Figure 1:**
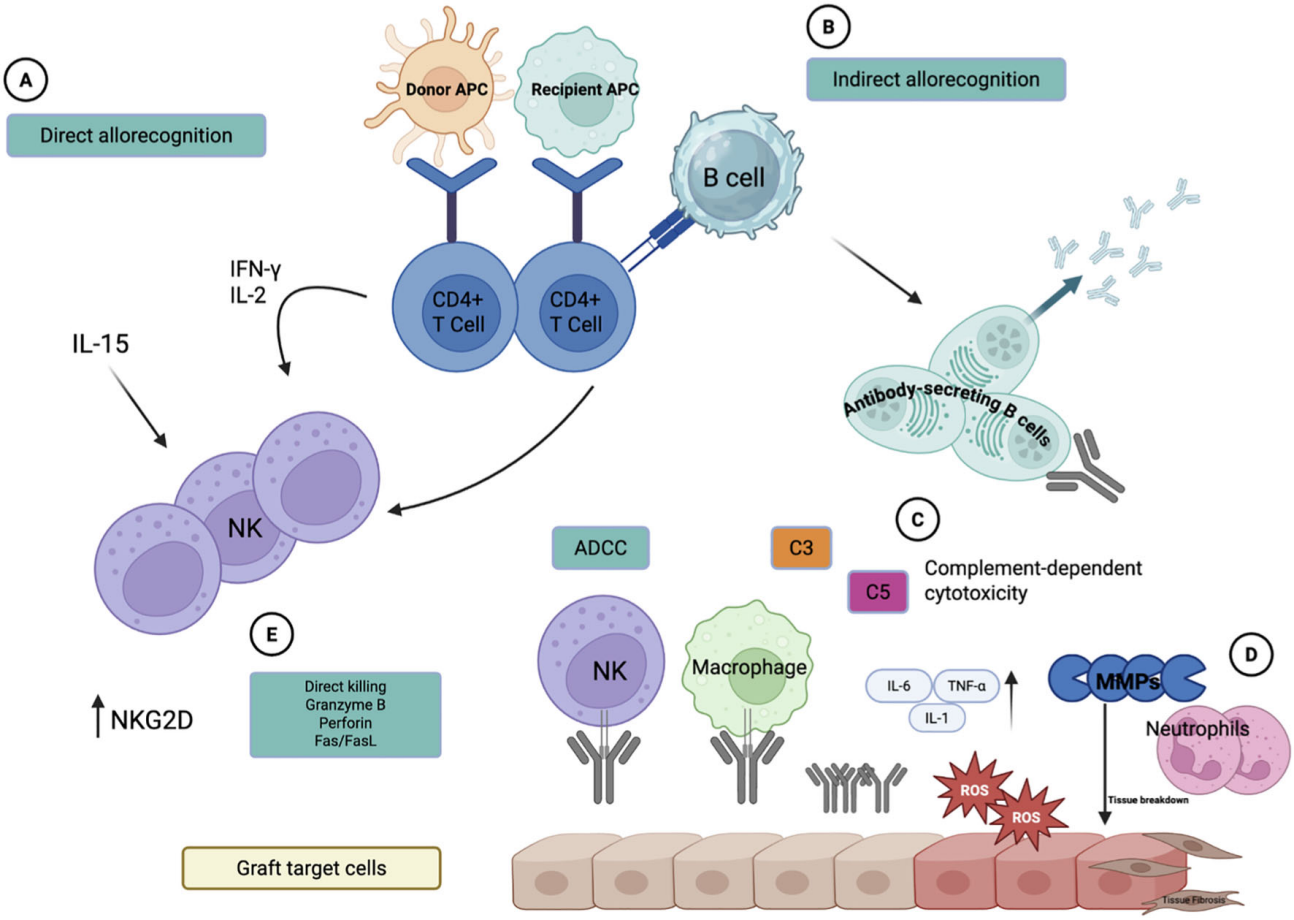
Immunologic pathways contributing to skin graft rejection. This figure illustrates the major pathways involved in graft rejection, including direct **(A)** and indirect **(B)** allorecognition, antibody-mediated complement activation **(C)**, and neutrophil and ROS mediated responses **(D)**. NK-cell mediated cytotoxic reaction is shown in **(E)**. The combined action complement, ROS, pro-inflammatory cytokines, and MMP lead to transition of a healthy graft epithelium to inflamed, damaged tissue. (Created with BioRender).

Graft injury during transplantation is not only mediated by immune cells but also by a cascade of chemical messengers which amplify tissue damage. In the beginning of the cascade, production of reaction oxygen species (ROS) released by activated neutrophils and macrophages in response to ischemia-reperfusion injury. These ROS can directly damage lipid membranes, proteins, and DNA, therefore, compromising graft integrity worsening inflammation (17). Excessive ROS generation can also contribute to upregulation of pro-inflammatory cytokines, creating a state of self-sustaining cycle of injury. Similarly, complement activation acts as another major contributor to graft rejection. Preformed alloantibodies bind donor antigens and trigger the classical complement pathway activation, bringing the formation of the membrane attack complex (MAC). This results in endothelial cell lysis, increased vascular permeability, and recruitment of leukocytes *via* C3a and C5a of complement, all which contribute to hyperactivity and acute rejection (18). Additionally, MMPs- a family of zinc-dependent endopeptidases- are released by both immune cells and graft parenchymal cells. MMPs degrade extracellular matrix proteins, promoting immune cell trafficking into the graft but also contributing to tissue breakdown and fibrosis if left unregulated. Specifically, MMP-2 and MMP-9 contribute to early graft remodeling and chronic rejection (19). (**Figure 1**).

These molecular mediators not only amplify immune damage but also interfere with revascularization, structural stability, and long-term graft survival, highlighting their critical role in the immunopathology of graft rejection (19). New evidence suggests that inflammation, while detrimental when excessive, can also function as an adaptable helper when correctly regulated, supporting angiogenesis, tissue remodeling, and immune-mediated tissue repair (20). Controlled activation of innate immune pathways like macrophages-driven vascular endothelial growth factor (VEGF) release, regulatory T cells (Treg) secreted cytokines, and low-grade ROS signaling all have been shown to promote neovascularization enhancing graft integration and regenerative healing (21). Macrophages play essential and sequential roles in wound repair, with classically activated macrophages involved in early inflammatory responses and alternative activated macrophages contributing to later tissue repair and angiogenesis (22). As inflammation resolves, repair promoting macrophages (M2) further promote fibroblast activation, extracellular matrix deposition, and long-term tissue remodeling (20). These findings support the rationale for emerging immunomodulatory therapies by emphasizing inflammation as a biologically adjustable process with therapeutic potential.

## 3 Immunomodulation and emerging therapies

### 3.1 Conventional immunosuppression and broad control of adaptive inflammation

Immunosuppressive pharmacotherapy is critical to prevent skin graft rejection, with agents targeting both innate and adaptive immune responses. Immunosuppressive agents like tacrolimus, corticosteroids, and cyclosporine, have demonstrated effectiveness in reducing T cell-mediated inflammation (**Table 1**) (23). Tacrolimus, a calcineurin inhibitor, acts by blocking T-cell activation therefore, inhibiting IL-2 transcription and has shown use in topical formulations for skin grafts and dermatoses alike (24). Systemic corticosteroids suppress both humoral and cellular immune responses and are often used preoperatively to improve graft survival, although long term use is limited by adverse effects such as infection and impaired wound healing (25). Additionally, because skin grafts are among the most immunogenic tissues, their rejection is often not adequately controlled by standard systemic immunosuppressive therapy, underscoring the need for strategies that directly reduce graft immunogenicity and improve long-term survival (26).

**Table 1:** Emerging strategies for controlling inflammation in skin graft survival.

Therapeutic strategy	Primary inflammatory target(s)	Mechanism of immune modulation
Systemic immunosuppressants (e.g., tacrolimus, corticosteroids)	T-cell activation, pro-inflammatory cytokines (IL-2, TNF-α)	Broad suppression of adaptive immune responses through inhibition of T-cell signaling and cytokine transcription
Biomaterial-based scaffolds and engineered skin substitutes	Antigen-presenting cells, reactive oxygen species (ROS), local cytokine gradients	Local immune regulation via reduced antigenicity, antioxidant incorporation, and controlled release of anti-inflammatory agents
Cytokine-targeted therapies (e.g., TNF-α, IL-1β, IL-6 inhibitors)	Pro-inflammatory cytokine signaling pathways	Selective blockade of inflammatory mediators to limit immune cell recruitment and tissue injury
Stem cell–based and stem cell–derived therapies (e.g., MSCs, conditioned media)	Macrophage polarization, T-cell activation	Promotion of regulatory immune phenotypes (M2 macrophages, Tregs) and suppression of pro-inflammatory cytokine production
Complement and innate immune pathway modulation	Complement cascade, NK cell activation, innate effector cells	Inhibition of early innate immune activation and ischemia–reperfusion–induced inflammation

### 3.2 Biomaterial-based strategies for local inflammation control

To overcome the limitations of systemic immunosuppression and improve long-term graft survival, recent advances have focused on developing anti-inflammatory and immuno-modulating agents that actively shape the local immune response. For instance, cellular and acellular skin substitutes are now being designed with scaffold materials, evasive immune coatings, and controlled drug-release systems that modulate the local immune response as well as oxidative stress levels at the graft site (**Table 1**). In one comprehensive study, the authors highlight a triad of strategies: first is the removal of donor antigen presenting cells (donor dendritic cells) prior to transplantation; second is the use of mesenchymal or other stem cell populations within the graft to allow immunoregulatory and regenerative cues; and the last factor is a scaffold design that incorporates anti-inflammatory molecules to act as antioxidants (27). In terms of tolerance induction, the review by Zhou et al. highlighted that donor-derived dendritic cells are key indicators of skin graft rejection and that reducing immunogenicity of the graft via depletion of key messenger molecules such as leukocytes, modulation of both direct and indirect allorecognition pathways, or promotion of regulatory T-cell development is crucial for long-term survival of skin allografts (26). From a biomedical perspective, several approaches have been proposed: for example, employing the biomimetic extracellular matrix scaffolds impregnated with anti-TNFα or anti-IL-6 agents; embedding antioxidant nanoparticles to reduce ROS at the implantation site as well as integrating the controlled-release systems which are able to deliver immunosuppressive factors locally rather than systematically (**Table 1**) (27). Regenerative-medicine frameworks further emphasize the importance of engineered constructs that combine bioactive scaffolds, stem-cell-derived keratinocytes or fibroblasts, and immunomodulatory agents to create a native dermal-epidermal barrier. Mahajan et al. highlights that tissue engineered skin substitutes have started to increasingly incorporate stem-cell populations and bioactive materials to reduce inflammatory cytokines, and increase re-epithelialization as well as graft tolerance in large deep wounds (28). Lastly, Arabi et al. describes how innate immune activation specifically from NK cells, macrophages, and early complement activation pathways remain the dominant barrier in xenogeneic skin grafting, promoting the development of immune-stealth biomaterials and genetically modified donor tissues designed to reduce cross-species antigenicity (28). Emerging xenotransplantation models have shown that modulating these innate immune responses through complement-inhibitory agents, macrophage suppressive drugs, or gene-edited donor tissues have the potential to delay rejection and could form the future design of allogeneic engineered skin constructs (29). These xenogeneic insights reflect a growing move toward immuno-engineered grafts that limit early innate rejection and promote regenerative repair.

### 3.3 Tissue engineering and immune-compatible skin substitutes

These innovations focus on minimizing the need for systemic immunosuppressive therapy like systemic glucocorticoids while enhancing tissue ability to regenerate and integrate, making them promising adjuncts in future clinical medicine. Recent discoveries in tissue engineering have allowed the emergence of immune-compatible skin substitutes designed to modulate inflammation and aid graft tolerance. One promising therapeutic approach is 3D bioprinting, which allows precise layering of keratinocytes, fibroblasts, and extracellular matrix components onto bioresorbable scaffolds that mimic native skin structure (30). Recent advances in the field of *in-situ* bioprinting further demonstrate the potential of depositing keratinocytes and dermal fibroblasts directly into wounds using portable bioprinter devices, allowing for rapid coverage and improved re-epithelialization in preclinical models (31). These constructs can be adjusted to include immune-evasive materials or controlled drug-release systems that mitigate inflammation and foster vascularization and improved tissue integration.

### 3.4 Targeting cytokine and oxidative stress pathways

An additional innovative approach involves the use of tissue-engineered grafts incorporating immune-modulating scaffolds, like those made from decellularized dermis or collagen- chitosan composites, which reduce antigenicity as well as supporting tissue healing. These scaffolds can be loaded with anti-inflammatory agents or growth factors which help wound healing without evoking immune rejection (32). Lastly, local delivery of cytokine inhibitor agents like IL-1β or TNF-α blockers as well as stem cell derived factors have demonstrated reduction in immune cell infiltration and oxidative stress generation in graft models (**Table 1**). For instance, mesenchymal stem cell (MSC) conditioned media can modulate macrophage polarization and suppress pro-inflammatory cytokines, enhancing graft take and tissue repair.(**Table 1**) (33) These approaches aim to reduce systemic immunosuppression while targeting immune microenvironments to be highly specific, potentially transforming graft design.

### 3.5 Translational challenges in immunomodulation

Despite the wealth of knowledge gained from animal models and preclinical systems, translating these findings into effective human therapies remains a significant challenge in skin graft immunology. Murine models have been pivotal in dissecting immune rejection pathways and testing emerging biomaterials or immunomodulatory therapies. However, these models lack immune complexity, skin structure, and comorbid variability present in human patients (8). In addition to these practical barriers, legal frameworks also require meticulous safety data, delaying the translation of promising biomaterials and cytokine- targeted therapies. As such, while experimental strategies show promise, clinical implementation requires cautious individual interpretation, improved humanized models, and collaborative efforts to design trails that reflect the real-world patient populations.

## 4 Limitations and challenges

Despite promising preclinical findings, multiple major obstacles prevent the effective translation of immunologic skin graft research into clinical practice. One of the most critical limitations is the difficulty in replicating the immune microenvironment observed in animal models to human subjects. Even though murine models are valuable for understanding the immunologic cellular and molecular pathways, they do not accurately represent human skin structure, immune cell diversity, or the heterogeneity of inflammatory responses in human patients (8). For example, differences in skin thickness, resident immune cell populations, as well as gene expression differences in humans result in varying outcomes that reduce predictive validity in clinical settings. For example, some of the differences include the fact that mice have thinner skin, different microbiota, as well as divergent immune gene expression, all which limit the predictive ability of therapeutic outcomes. One of the main challenges is being able to standardize patient selection due to heterogeneity in immune status, wound type, and co-existing conditions like diabetes or immunosuppression. Moreover, long-term graft monitoring, which is easily conducted in laboratory settings, is often logistically unfeasible or cost-prohibitive in clinical trials (34). Clinical trials attempting to bridge this gap are limited. However, multiple efforts are being made like the application of mesenchymal stem cell-conditioned media have shown some translational promise in modulating graft-associated inflammation in human subjects (35). Moreover, therapeutic strategies face the challenge of balancing the suppression of harmful inflammation without impairing wound healing. Systemic immunosuppressants like corticosteroids and calcineurin inhibitors can reduce immune rejection but at the same time delay tissue regeneration and increase risk for infection. Clinical attempts to use local therapies like tacrolimus or cytokine inhibitors remain limited, and maintaining this balance has been challenging (24, 25). Lastly, regulatory and ethical concerns in translational immunology further delay the clinical application of these emerging novel therapies. Human trials require cautious safety profiling as well as long-term monitoring, which are all difficult to pursue given the complexity of graft rejection and the chronicity of immune responses. Ethical concerns also arise when testing biomaterials or gene-edited constructs in vulnerable patient populations. These issues highlight the need for improved humanized models and collaborative research frameworks to design more predictive and ethically sound trials (34).

## 5 Discussion

The immunologic response to skin grafts involves a dynamic interplay between innate and adaptive immunity, with each system contributing distinctly to grafts fate. Innate immunity starts the inflammatory cascade by activation of neutrophils, macrophages, and complement system, rapidly responding to graft injury and ischemia-reperfusion damage. Reactive oxygen species (ROS) and pro-inflammatory cytokines such as TNF-α and IL-1β function as double-edged swords; they are crucial for the initial defense of the host but can become harmful when produced in excess, leading to graft rejection (19). Meanwhile, the adaptive immune system drives graft rejection primarily through alloantigen recognition pathways, involving CD4+ T and CD8+ T cells and downstream cytokine secretion. This progression from innate signal amplification to adaptive cytotoxicity highlights the need for interventions that can impact both aspects of the immune system. Emerging therapeutic agents offer hope for precise immunomodulation. Localized delivery of immunosuppressants such as topical tacrolimus and stem cell-derived factors have shown efficacy in controlling inflammation without systemic side effects (24). Bioengineered scaffolds incorporating decellularized dermis or cytokine inhibitors (e.g., IL-1β antagonists) present another promising frontier, aiming to reduce antigenicity while promoting tissue integration (36). However, questions remain regarding the durability, safety, and regulatory approval of these innovations. Most preclinical studies are limited by short follow-up periods and lack robust human data while only a few clinical trials are currently in phase I/II. Additionally, numerous efforts to induce tolerance to allogenic skin graft without the use of immunosuppression have failed likely due to the strength and complexity of immune response associated with such transplant. Translational research stands as a cornerstone for bridging laboratory insights and clinical reality. The immunologic discrepancies between animal models and human patients underscore the urgency for better humanized models and individualized trial designs. For example, efforts like human skin xenografts in immunodeficient mice or 3D bio printed constructs using patient-derived cells may improve relevance and predictive accuracy (23). Furthermore, collaborative multicenter trials with stratified patient cohorts may help overcome the challenge of clinical heterogeneity in wound types and immune status. Ultimately, closing this translational gap will require not only scientific innovation but also infrastructure support, ethical frameworks, and long-term investment in immune-engineered solutions.

Taken together, current evidence demonstrates that inflammation exerts a dual-edged effect on skin graft outcomes. On one hand, controlled inflammatory signaling is essential for initiating tissue repair, promoting angiogenesis, and activating regenerative pathways that support graft take and integration. On the other hand, unchecked or misdirected immune activation- driven by innate effectors like neutrophils and macrophages or adaptive responses mediated by T and B lymphocytes- can result in graft rejection through direct cytotoxicity, complement activation, and fibrosis. This dual role underscores the critical importance of fine-tuned immune modulation. Current therapeutic strategies, including systemic immunosuppressants and bioengineered scaffolds, offer some success but are limited by systemic toxicity, variable patient response, and lack of long-term efficacy data. Future research must prioritize the development of localized, biomaterial-based interventions that modulate immune responses at the graft site without compromising systemic immunity. Moreover, advancing translational models that better recapitulate human immunobiology- such as humanized mouse models, 3D bio printed skin constructs, and patient-derived tissue systems- will be vital to overcoming the limitations of current preclinical tools.

To truly optimize graft survival, interdisciplinary efforts are needed to integrate immunology, biomaterials science, and clinical research. Investment in longitudinal clinical trials, personalized immunotherapy, and real-time graft monitoring tools will enable a new era of precision grafting. Additionally approaches such as blockage of interleukin-6 (IL-6) signaling with agents like tocilizumab has shown clinical benefit in conditions with immune dysregulation and inflammation, highlighting the translational potential of targeted cytokine inhibition to control excessive inflammatory signaling (37). Similarly, agents like afimkibart, a TNFSF15/TL1A neutralizing antibody, are being explored for chronic inflammatory bowel diseases, demonstrating how modulation of novel cytokine pathways can suppress pathological inflammation (38). Ultimately, in future embracing inflammation as a controllable ally- rather than an inevitable threat- may hold the key to transforming outcomes in skin grafting and reconstructive medicine.
